# Tousled kinase TLK1B counteracts the effect of Asf1 in inhibition of histone H3–H4 tetramer formation

**DOI:** 10.1186/1756-0500-2-128

**Published:** 2009-07-08

**Authors:** Arrigo De Benedetti

**Affiliations:** 1 Department of Biochemistry and Molecular Biology and the Feist-Weiller Cancer Center, Louisiana State University Health Sciences Center, Shreveport, LA 71130, USA

## Abstract

**Background:**

The *T*ousled-*l*ike *k*inases (TLKs) function in processes of chromatin assembly, including replication, transcription, repair, and chromosome segregation. TLK1 interacts specifically with the chromatin assembly factor Asf1, a histone H3–H4 chaperone, and with Rad9, a protein involved in DNA repair. Asf1 binds to the H3–H4 dimer at the same interface that is used for formation of the core tetramer, and hence Asf1 is implicated in disruption of the tetramer during transcription, although Asf1 also has a function in chromatin assembly during replication and repair.

**Findings:**

We have used protein crosslinking with purified components to probe the interaction between H3, H4, Asf1, and TLK1B. We found that TLK1B, by virtue of its binding to Asf1, can restore formation of H3–H4 tetramers that is sterically prevented by adding Asf1.

**Conclusion:**

We suggest that TLK1B binds to Asf1 in a manner that interferes with its binding to the H3–H4 dimer, thereby allowing for H3–H4 tetramerization. A description of the function of TLK1 and Asf1 in chromatin remodeling is presented.

## Background

The gene *Tousled *of *Arabidopsis thaliana *encodes a protein kinase which, when mutated, results in abnormal flower development [1], possibly from failure to protect the genome from UV damage [2,3] and resulting in mitotic aberrations [4,5]. Alternatively, the defects may involve other activities of Tousled like kinases (TLKs) in transcription [6], or in segregation of chromosomes at mitosis [4,7]. Several physiological substrates of *Tousled *kinases have been identified, namely Asf1 [8], histone H3 [9], and more recently Rad9 [10], which suggested a function in chromatin assembly [11] with implications in transcription [12,2], condensation of chromosomes [4], and DNA repair [3,13]. TLK1 is generally considered a gene of metazoans, although TLK1 is also present in trypanosomes [14]. In all mammals studied, the primary TLK1 transcript is alternatively spliced to two main isoforms [15,9] termed TLK1 and TLK1B, which probably have very similar function [10], and that we often refer to as TLK1/1B. TLK1/1B binds and phosphorylates Asf1 in all organisms in which it is found, with the exception of yeast [8], despite the conservation of Asf1 among all species.

Asf1 is a histone H3–H4 chaperone [16] that is essential in mammalian cells [17] and other organisms [18] but not in yeast, although yeast cells deleted for Asf1 are sensitive to genotoxins [19]. Asf1, in conjunction with CAF1, promotes the assembly of nucleosomes onto newly replicated DNA, but it can also promote nucleosome eviction at activated promoters [20,21]. Thus, Asf1 is generally involved in chromatin remodeling, that also entails DNA repair [19,22]. The crystal structure of Asf1 in complex with H3–H4 was solved at high resolution [23], and Asf1 was found to cover the dorsal site of the H3–H4 dimer, thereby sterically preventing formation of the core tetramer. This is thought to be important for disrupting nucleosomes during transcription or remodeling of chromatin in damaged DNA [3,10].

## Findings and Discussion

**Figure 1:**
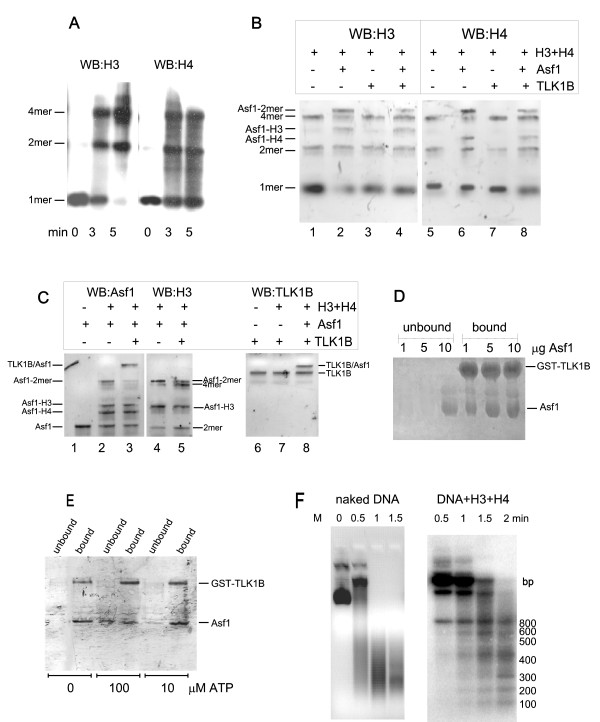
**A. Crosslinking of H3–H4**. Histones H3 and H4 were incubated for the indicated minutes and then crosslinked with formaldehyde before separation on a 15% SDS/PAGE. The blot was probed separately with anti-H3 and anti-H4. B. Effect of Asf1 and TLK1B on H3–H4 dimer and tetramer formation. Reactions containing the indicated combinations of H3, H4, Asf1, and TLK1B (all in equivalent amount) were crosslinked as described in Methods and immunoblotted for H4 or H3. C. Western blots for Asf1 and TLK1B. The indicated reactions as in panel B were run in duplicate lanes and immunoblotted for Asf1, H3, or TLK1B (the gel was run for a longer time than in panel B to separate the larger proteins). For antibody controls, lane 1 contained only Asf1, and lane 7 contained only TLK1B. The positions of the cross-linked complexes identified by mobility and immunoreactivity are indicated. D. TLK1B and Asf1 bind each other stoichiometrically. Reactions contained 5 μg of GST-TLK1B and varying amounts of Asf1, as indicated. After 10 min at room temperature, the samples were adsorbed on GSH-Sepharose and analyzed for bound and unbound fractions after separation on a 10% SDS/PAGE, which was stained with Coomassie blue. E. Interaction of TLK1B and Asf1 and the effect of ATP. GST-TLK1B and Asf1 (1 μg each) were incubated for 10 min at room temperature with and without 10 or 100 μM ATP, before analysis by GSH-Sepharose pull-down. The 10% SDS/PAGE gel was stained with Coomassie blue. F. MNase digestion of pBluescript assembled into pseudo-nucleosomes. In the left panel, naked supercoiled plasmid was digested with MNase for the indicated time. In the right panel, the plasmid was first incubated with equimolar H3 and H4 in high salt and then step-dialyzed as described in [3], before MNase digestion. The DNA was re-extracted from the reactions with Geneclean and run on a 1.5% agarose/TAE gel. The resulting ~120 bp ladder (a bit shorter than the repetitive 146 bp of nucleosomal DNA) is indicative of formation of a chromatinized template. The positions of the bands of a 100-bp ladder (GenRuler, Fermentas) are indicated.

To test the effect of Asf1 and TLK1B on formation of H3–H4 dimers and tetramers, conditions were first optimized for the time of assembly and method of crosslinking with formaldehyde. Figure 1A shows the formation of the cross-linked H3–H4 dimer and tetramer during a time course of assembly at room temperature, as described in Methods. The gel was blotted and probed with H3 or H4 antiserum.

Figure 1B shows the effect of Asf1 and TLK1B on formation of the H3–H4 tetramer. The addition of Asf1 reduced the assembly of H3–H4 tetramer (see lane 2 or 6), with respect to the H3+H4 lane alone (lane 1 and 5), consistent with the steric hindrance mechanism proposed by others [24,23]. Quantitation of the tetramer from three independent reactions and several blots showed that Asf1 inhibits tetramer formation by 57%. The addition of TLK1B negates the effect of Asf1 on inhibition of tetramer formation (lane 4 and 8) and restores normal levels of the tetramer, but apparently does not prevent formation of the Asf1-H3 and Asf1-H4 complex. The co-crystal structure revealed that Asf1 has independent binding sites for H3 and H4 [23]. The addition of TLK1B does inhibit the formation of the Asf1-H3/H4 heterotrimer (Asf1-2mer; compare lanes 2 and 6 with lanes 4 and 8). These data support a model in which TLK1B alters the association of Asf1 with the H3–H4 dimer, which may be significant for the reformation of core tetramers, for example during reassembly of nucleosomes at the repaired junctions of a DSB [10], or in attenuation of transcription [21]. This is critically important since it was postulated that essentially all the chromatin-unbound fraction of H3–H4 dimer is associated with Asf1 [23], and hence, unavailable for nucleosome assembly. It is noteworthy in this context that TLK1 is rather abundant, at least in mammalian cells, and that TLK1B is further increased after genotoxic stress [10] reaching levels that may significantly affect the interaction of Asf1 with the H3–H4 dimer. Indeed, TLK1B was previously shown to promote chromatin assembly in vitro when Asf1 was also present [3,10].

To further characterize the composition of the cross-linked proteins in the reactions assembled in panel B, selected lanes were analyzed in parallel blots that were probed for Asf1 and TLK1B, and again H3 for alignment of the bands (panel C). Thus the various protein complexes were identified by probing with all 4 antibodies and by mobility. Although the stoichiometric representation of the cross-linked proteins is difficult to assess by western blot, ~50% of the input TLK1B (shown in lane 7) became crosslinked to the input Asf1 (lanes 3 and 8).

To better determine the stoichiometry of TLK1B-Asf1 complexes, 5 μg of GST-TLK1B was incubated with varying amounts of Asf1, and their association determined by GSH-Sepharose pull-down (panel D). From this experiment, TLK1B and Asf1 appear to bind at a 1:1 ratio, in the absence of other competing interactions (e.g., H3 and H4). So far, all the reactions were carried out in the absence of ATP, so that it is the chaperone function of TLK1B [10] and not its kinase activity that was tested.

To probe the role of the kinase activity of TLK1B (i.e., the phosphorylation of Asf1) on the association of the two proteins, ATP was added to the reactions. It was previously demonstrated that TLK1 binds, phosphorylates, and subsequently releases Asf1 *in vitro *[8]. When a kinase-dead mutant of TLK1 was used, Asf1 remained bound to TLK1, suggesting that phosphorylation of Asf1 reversibly modulates the interaction between the two proteins [8]. In previous work we also found that TLK1B required ATP concentrations above 10 μM (measured by γ32P-ATP trace labeling), so ATP was added at 10 and 100 μM ATP. The two proteins were incubated as in panel D and analyzed by pull-down. ATP decreased the retention of Asf1 (via binding to GST-TLK1B) on the resin (panel E), which was detected in the unbound fraction, presumably due to the dissociation of the TLK1B-Asf1 complex after phosphorylation of Asf1. How the addition of ATP may affect the equilibrium of H3–H4 dimers and tetramers in a reaction assembled as in panel B lane 4 is certainly something worth investigating further. But importantly, TLK1B also phosphorylates H3 at S10, which could also affect the dimer/tetramer equilibrium and complicate the interpretation of such an experiment. Furthermore, the cross-linking is really not designed to reveal subtle differences in equilibrium formation of these complexes, as this would require more sophisticated biophysical methods.

A possible concern of the foregoing analysis is that formation of H3–H4 cross-linked complexes is simply due to aggregation that resulted from improperly folded proteins. To address this concern, a chromatin assembly assay on a circular plasmid was carried out. H3–H4 core tetramers assemble on DNA in a salt dilution assay forming a basic structure similar to nucleosomes [25]. Thus, chromatin formation is indicative of a correct assembly of H3 and H4 in the core tetramer. Panel F shows that in a MNase susceptibility assay, a ~120 bp ladder was generated, which is a hallmark of H3–H4 tetramer assembly on a DNA templeate [25].

In conclusion, these in vitro experiments indicate that TLK1/1B can act as a chaperone (or buffer/regulatory factor) for Asf1. One might imagine that this would be especially important under conditions when Asf1 may be recruited at specific chromosomal locations to disrupt the H3–H4 tetramer, resulting in nucleosome eviction. TLK1/1B reduces the interaction of Asf1 with the H3–H4 dimer (at least in the experiments performed here), thus shifting the equilibrium toward formation of the core tetramer. A further complexity of this model is the possible role of phosphorylation of TLK1 at S695 via ATM, which inhibits its kinase activity [26]. We postulate that the resulting reduction of Asf1 phosphorylation would result in a more stable association of the TLK1-Asf1 dimer, instead of a kinetic association between the two proteins involving the ratio of unphosphorylated and phosphorylated Asf1. Hence, the phosphorylation/inactivation of TLK1, for example after DNA damage, would restrict the availability of Asf1 to act as a H3–H4 chaperone. In fact, this may result in release of the H3–H4 dimer from Asf1, and hence an increase in the available pool for tetramer formation. Increased H3–H4 tetramer could impact transcription, or replication and DNA repair, where the H3–H4 tetramer is probably re-deposited onto DNA by other H3–H4 chaperones such as the HIR complex [24] or RSC in the case of DSB repair [27].

## Materials and methods

Recombinant human histone H4 was purchased from NE Biolabs and bovine H3 from Roche. Recombinant human TLK1B and Asf1B were prepared as described previously [10]. H3 and H4 antisera were from Upstate biotechnology. Asf1 antiserum was from Santa Cruz Biotechnology. TLK1 antiserum was prepared in our lab [9]. Reactions (0.1 ml in 50 mM Phosphate buffer, pH7.2) were assembled on ice and then incubated for 5 min (unless otherwise stated) at room temperature (RT). Crosslinking was performed with 3 mM formaldehyde for 5 minutes on ice, and the reaction was stopped by the addition of 50 mM glycine. The samples were boiled for 1 min in Laemlli's sample buffer and separated on 10 or 15% PAGE/SDS. After immunoblotting the membranes were developed with Opti4-CN (Promega). The TLK1B/Asf1 pull-down on GSH-Sepharose was as described in [10]. For these pulldowns, the two proteins were reacted for 10 min at RT, with and without ATP (and 1 mM MgCl_2_). Chromatin assembly with H3 and H4 (10 μg each) was carried out on 4 μg of pBluescript in 1.5 M NaCl, which was then step-dialyzed before MNase digestion, as described in [3].

## Competing interests

The author declares that they have no competing interests.

## Authors' contributions

ADB is solely responsible for this article
